# Utilization of standardized preimplantation genetic testing for aneuploidy (PGT-A) via artificial intelligence (AI) technology is correlated with improved pregnancy outcomes in single thawed euploid embryo transfer (STEET) cycles

**DOI:** 10.1007/s10815-022-02695-7

**Published:** 2023-01-07

**Authors:** Julia Buldo-Licciardi, Michael J. Large, David H. McCulloh, Caroline McCaffrey, James A. Grifo

**Affiliations:** 1 New York University Grossman School of Medicine, 550 First Avenue, NBV 9E2, New York, NY 10016 USA; 2 CooperSurgical, Inc., 75 Corporate Drive, Trumbull, CT 06611 USA; 3 New York University Langone Fertility Center, 159 E 53rd Street 3rd Floor, New York, NY 10022 USA

**Keywords:** Pre-implantation genetic testing, Artificial intelligence, Mosaicism, In vitro fertilization, Single thawed euploid embryo transfer

## Abstract

**Purpose:**

To investigate the role of standardized preimplantation genetic testing for aneuploidy (PGT-A) using artificial intelligence (AI) in patients undergoing single thawed euploid embryo transfer (STEET) cycles.

**Methods:**

Retrospective cohort study at a single, large university-based fertility center with patients undergoing in vitro fertilization (IVF) utilizing PGT-A from February 2015 to April 2020. Controls included embryos tested using subjective NGS. The first experimental group included embryos analyzed by NGS utilizing AI and machine learning (PGTai^SM^ Technology Platform, AI 1.0). The second group included embryos analyzed by AI 1.0 and SNP analysis (PGTai2.0, AI 2.0). Primary outcomes included rates of euploidy, aneuploidy and simple mosaicism. Secondary outcomes included rates of implantation (IR), clinical pregnancy (CPR), biochemical pregnancy (BPR), spontaneous abortion (SABR) and ongoing pregnancy and/or live birth (OP/LBR).

**Results:**

A total of 24,908 embryos were analyzed, and classification rates using AI platforms were compared to subjective NGS. Overall, those tested via AI 1.0 showed a significantly increased euploidy rate (36.6% vs. 28.9%), decreased simple mosaicism rate (11.3% vs. 14.0%) and decreased aneuploidy rate (52.1% vs. 57.0%). Overall, those tested via AI 2.0 showed a significantly increased euploidy rate (35.0% vs. 28.9%) and decreased simple mosaicism rate (10.1% vs. 14.0%). Aneuploidy rate was insignificantly decreased when comparing AI 2.0 to NGS (54.8% vs. 57.0%). A total of 1,174 euploid embryos were transferred. The OP/LBR was significantly higher in the AI 2.0 group (70.3% vs. 61.7%). The BPR was significantly lower in the AI 2.0 group (4.6% vs. 11.8%).

**Conclusion:**

Standardized PGT-A via AI significantly increases euploidy classification rates and OP/LBR, and decreases BPR when compared to standard NGS.

## Introduction

Pre-implantation genetic testing for aneuploidy (PGT-A) has evolved over the last 28 years to become the most accurate method to assess embryo chromosomal competency. Next generation sequencing (NGS) is the most widely used platform for PGT-A, sequencing millions of small DNA fragments in parallel and mapping them to the human reference genome by bioinformatics analyses [1]. This technology, when performed with sufficient resolution, can identify unbalanced translocations, segmental aneuploidy, and some triploidy more sensitively than its precursor array comparative genome hybridization (aCGH). With high levels of sensitivity and demonstrated linearity in detection, NGS has also increased the ability to detect embryo biopsy mosaicism, enhancing identification of embryos with increased or reduced viability [2].

Despite initial NGS methods improving the analytical accuracy of PGT-A, its limitations include the inability to detect all forms of polyploidy such as 69, XXX, and the inability to identify maternal vs. paternal sources of aneuploidy. In addition, there remains room for improvement in the designation of embryos as suitable for transfer based on mosaic characterization [3]. Refining the ability of NGS to discriminate between euploid and non-euploid embryos is essential in advancing prediction models and PGT-A as a useful clinical tool.

Artificial intelligence (AI) harnesses mathematical algorithms and machine learning technology to decrease human subjectivity, enhance the differentiation of true signal from noise, and maximize sensitivity and specificity of embryo classification [4]. Here, we describe embryo biopsy, transfer and pregnancy outcomes of in vitro fertilization (IVF) cycles with PGT-A using an artificial intelligence platform with two independent methods for aneuploidy detection, including normalized read quantification for copy number variation and single nucleotide polymorphism analysis. This platform also detects haploidy as well as both male and female triploidy. Together, these technological features may serve to improve resolution of mosaicism and ploidy status in trophectoderm biopsies and concomitant embryo classification.

The purpose of this study was to assess the embryo classification rates using standardized PGT-A algorithms and evaluate the impact those classifications have on single thawed euploid embryo transfer (STEET) clinical outcomes. We hypothesized that technological advancements improving signal-to-noise processing would decrease over-interpretation of molecular noise and increase the sensitivity of small signal changes, thereby decreasing biochemical pregnancy rates and spontaneous abortion rates, and increasing implantation rates, clinical pregnancy rates and ongoing pregnancy/live birth rates.

## Materials and methods

### IVF patient protocols

All patients underwent controlled ovarian hyperstimulation (COH) using recombinant follicle-stimulating hormone (FSH) or a combination of recombinant FSH with human menopausal gonadotropins (hMG). Lutenizing hormone suppression was performed using either gonadotrophin-releasing hormone (GnRH) antagonist starting once follicular diameter achieved 13 mm or a GnRH agonist in a long or short protocol. Ovulation was triggered with either 10,000 IU of hCG or a combination of 40 U of GnRH agonist and 1,000 IU of hCG when lead follicles reached 18–20 mm. Oocytes were retrieved 35 hours following trigger and fertilized using standard insemination when possible.

Intracytoplasmic sperm injection (ICSI) was performed for cases when the total motile counts recovered following semen processing were less than 2 million or for patients with a history of poor fertilization. Embryos were cultured using single step embryo culture medium (Global Embryo Culture Medium, LifeGlobal, LGGG 050) with 10% (v/v) protein supplement (LG protein supplement, Life Global, PGPS 050) to the blastocyst stage and underwent assisted hatching on day 4 followed by trophectoderm biopsy on days 5, 6, or 7.

### Preimplantation genetic testing

All PGT-A was conducted using a commercially available Genomics Testing Service Lab (CooperSurgical, Inc., Livingston, NJ).

All trophectoderm biopsies were lysed and amplified using the SurePlex™ DNA Amplification System (Illumina, Inc., San Diego, CA) prior to library preparation using VeriSeq Library Preparation Kit-PGS or Nextera XT (Illumina, Inc.).

Subjective NGS was conducted as described previously using VeriSeq PGS (Illumina, Inc., San Diego, CA) [4]. Briefly, libraries were sequenced on a MiSeq™ (Illumina, Inc.) with a single-end 1 × 36 base pair strategy targeting 1 M raw reads (36 M base pairs). Sequencing data were normalized and converted into plots using BlueFuse™ Multi Software followed by manual assessment performed by competent laboratory staff who completed didactic training, statement of purpose review, observation, supervised evaluation, and blinded performance against known samples. Copy number plots were interpreted using the following definitions: < 20% deviation from 2.0 copies (1.8–2.2) as euploid, > 80% deviation from 2.0 copies (> 2.8 or < 1.2) as aneuploid, and between 20 and 80% deviation from 2.0 copies (1.2–1.8, 2.2–2.8) as mosaic. Embryos containing three or more mosaic chromosomes were classified as aneuploid.

AI 1.0 (PGTai^SM^ Technology Platform, CooperSurgical, Inc.) interprets sequencing data (produced as above) with a proprietary algorithm stack (test samples interrogated using models built using instance-based machine learning, linear regression, hidden Markov models, convolutional neural networks, etc. via Pandas, Scikit-learn, TensorFlow, etc.). Machine learning and statistical algorithms were trained using > 1,000 sequenced biopsies from embryos that led to pregnancies with no obvious genetic defects (representing true negative) and > 250 sequenced biopsies containing known unbalanced structural rearrangements (representing true positive). Independent biopsies of known and varied genetic makeup were then used to validate accurate performance (unpublished data, CooperSurgical, Inc.). The clinical pipeline first automatically calls any region that statistically deviates from reference populations, and then flagged regions are automatically interpreted with the same classification thresholds (20–80% is mosaic) as above, resulting in human-readable text results.

AI 2.0 (PGTai 2.0, CooperSurgical, Inc.) is a subsequent update to PGTai utilizing sequencing on a NextSeq™ (Illumina, Inc.) with a paired-end 2 × 36 base pair strategy targeting 4 M raw reads (288 M base pairs). Secondary modules were developed and validated (as described above but also utilizing SNP array data as truth) to interrogate drift in heterozygous SNP ratios as an aggregate signature across CNV regions called by the base algorithms, thereby verifying or rejecting lower confidence called CNVs. Genome-wide heterozygous SNP ratio drift was also assessed to classify 23,X haploid and 69,XXX triploid samples.

### Retrospective study

This retrospective cohort study was approved by the Institutional Review Board at New York University School of Medicine (NYU IRB # 13–00,389). Archived data were examined for in vitro fertilization (IVF) retrievals utilizing PGT-A and STEET from February 2015 to April 2020 at a single large university-based fertility center. Cohorts were grouped from periods of February 2015–October 2018, October 2018–October 2019, and October 2019–April 2020, and biopsies were tested using subjective NGS (control group), AI 1.0, or AI 2.0, respectively.

### Primary outcomes: embryo diagnostics

Primary outcomes included rates of euploidy, aneuploidy, and simple mosaicism for each PGT-A technology. Outcomes were also stratified into SART age ranges: < 35, 35–37, 38–40, 41, 42, and > 42. Classification rates were determined as follows:

Euploid rate = average number of euploid embryos/total embryos per retrieval.

Aneuploid rate = average number of aneuploid embryos/total number of embryos per retrieval.

Simple mosaicism rate (up to 2 mosaic chromosomes per embryo) = average number of single + double mosaics/total number of embryos.

Triploid rates (stratified by 69,XXX and 69,XXY) = total triploid embryos/number of embryos biopsied.

### Secondary outcomes: post embryo transfer pregnancy indices

Secondary outcomes included rates of implantation (IR: number of positive human chorionic gonadotropin [hCG]/total embryos transferred), clinical pregnancy (CPR: number of gestational sacs visualized on transvaginal ultrasound/total embryos transferred), biochemical pregnancy (BPR: number of losses per positive hCG without sac visualization), spontaneous abortion (SAR: pregnancy failure after a previously documented gestational sac/total number of clinical pregnancies), and ongoing pregnancy and/or live birth (OP/LBR: number of pregnancies maintained beyond twenty weeks gestation and/or live births/total embryos transferred). Outcomes were stratified into the SART age ranges described above. Monozygotic twins resulting from transfer of one embryo were counted as one implantation and one ongoing pregnancy and/or live birth.

### Statistical analysis

Aggregate PGT classification rates were assessed using a one-way ANOVA followed by Bartlett’s test using the NGS group as the control group. SART age group stratified data were assessed using two-way ANOVA followed by Dunnett’s multiple comparison test with individual variances using the subjective NGS group as the control comparison. Secondary outcomes were assessed using Fisher’s exact test. *p* values less than 0.05 were considered significant. Analyses were done using GraphPad Prism 9.2.0 for Windows, (GraphPad Software, San Diego, California).

## Results

### Primary outcomes

A total of 4,765 retrieval cycles including 24,908 embryos utilized PGT-A. A total of 97 retrieval cycles were eliminated due to incomplete data. The average patient age was 37.7 ± 4.2 years. A comparison of test platforms across all age groups can be found in Table 1, while rates of euploidy, aneuploidy, and simple mosaicism stratified by SART age categories can be found in Table 2.

**Table 1:** Aggregate study data comparing patient age, euploid rate, aneuploid rate, and simple mosaic rate between subjective NGS and AI 1.0 or AI 2.0. Listed confidence intervals describe the differences in AI means vs. the subjective NGS mean

	Subjective	AI 1.0	*p*	95% CI	AI 2.0	*p*	95% CI
Age	37.8 ± 4.2	37.3 ± 4.1	0.0065	− 0.8–0.1	37.5 ± 4.0	0.1368	− 0.7–0.1
Euploid	28.9%	36.6%	< 0.0001	5.2–10.2%	35.0%	< 0.0001	3.3–8.9%
Mosaic	14.0%	11.3%	0.0013	− 4.5–0.9%	10.1%	< 0.0001	− 5.9–1.9%
Aneuploid	57.0%	52.1%	0.0003	− 7.8–2.0%	54.8%	0.2381	− 5.4–1.0%

**Table 2:** Study data comparing patient age, euploid rate, aneuploid rate and simple mosaic rate between subjective NGS and AI 1.0 or AI 2.0 using SART age group stratification. Listed confidence intervals describe the differences in AI means vs the subjective NGS mean

Maternal age (yr)	Platform	Cases (*n*)	Euploid (%)	*p*	95% CI	Aneuploid (%)	*p*	95% CI	Simple mosaic (%)	*p*	95% CI
< 35	Subjective	715	43.6	-		36.2	-		20.2	-	-
	AI 1.0	221	53.3^*^	< 0.001	0.051–0.142	31.1^*^	0.047	− 0.101–0.001	15.6^*^	0.004	− 0.079–0.013
	AI 2.0	151	51.3^*^	0.002	0.025–0.129	33.8	0.588	− 0.083–0.035	14.9^*^	0.005	− .092–0.014
35–37	Subjective	693	35.6	-		47.2	-		17.2	-	-
	AI 1.0	170	41.9^*^	0.001	0.013–0.113	42.8	0.152	− 0.100–0.012	15.3	0.440	− 0.056–0.018
	AI 2.0	155	46.6^*^	< 0.001	0.058–0.162	40.4^*^	0.018	− 0.126–0.010	13.0	0.300	− 0.081–0.003
38–40	Subjective	885	25.6	-		61.8	-		12.7	-	-
	AI 1.0	237	34.5^*^	< 0.001	0.046–0.132	57.4	0.078	− 0.092–0.004	8.2^*^	0.003	− 0.077–0.013
	AI 2.0	159	30.5	0.058	− 0.001–0.010	60.1	0.722	− 0.074–0.038	9.5	0.108	− 0.069–0.005
41	Subjective	317	16.2	-		74.7	-		9.2	-	-
	AI 1.0	67	23.4	0.101	− 0.011–0.157	69.7	0.412	− 0.144–0.044	6.7	0.601	− 0.087–0.037
	AI 2.0	74	20.6	0.345	− 0.031–0.119	73.6	0.947	− 0.095–0.073	5.8	0.318	− 0.090–0.053
42	Subjective	252	13.4	-		80.8	-		5.9	-	-
	AI 1.0	56	8.9	0.426	− 0.131–0.041	87.1	0.268	− 0.034–0.160	4.0	0.756	− 0.083–0.045
	AI 2.0	37	14.9	0.934	− 0.088–0.118	81.4	0.991	− 0.109–0.121	3.6	0.749	− 0.099–0.053
> 42	Subjective	362	17.5	-		73.5	-		9.0	-	-
	AI 1.0	72	12.0	0.114	− 0.131–0.041	79.7	0.194	− 0.023–0.147	8.3	0.952	− 0.063–0.049
	AI 2.0	55	3.7^*^	< 0.001	− 0.223–0.053	95.2^*^	< 0.001	0.122–0.313	1.2^*^	0.012	− 0.141–0.015

^*^p < 0.05 compared to subjective

**Fig. 1:**
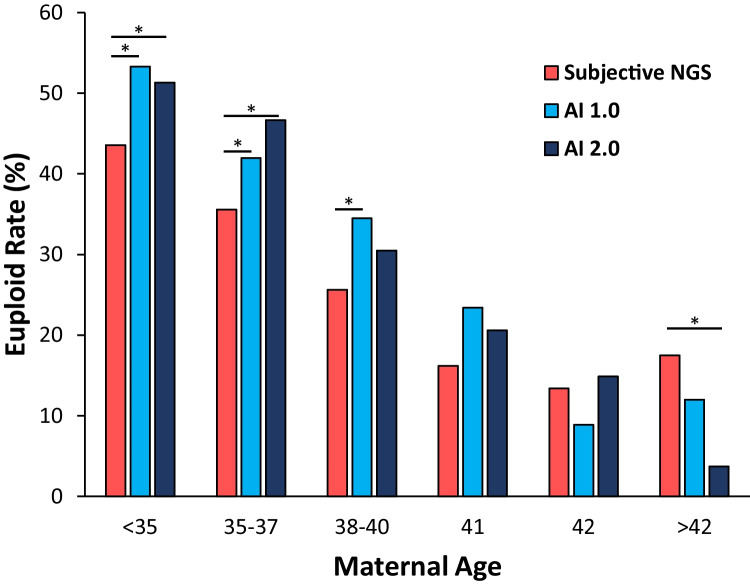
Euploidy rates by subjective NGS vs. AI 1.0 and subjective NGS vs. AI 2.0. ^*^p < 0.05 vs. subjective NGS

Overall, the euploidy rate was significantly higher when comparing AI 1.0 to subjective NGS (36.6% vs. 28.9%, *p* < 0.001). When stratified by SART ages, euploidy rates reached significance in the < 35, 35–37, and 38–40 old categories when comparing AI 1.0 to subjective NGS (Fig. 1). The euploidy rate was higher when comparing AI 1.0 to subjective NGS in the 41-year-old category but was not found to be significant. Euploidy rates were decreased when comparing AI 1.0 to subjective NGS in the 42 and > 42-year-old categories but lacked sufficient cohort sizes. Overall, the euploidy rate was also significantly higher when comparing AI 2.0 to subjective NGS (35.0% vs. 28.9%, *p* < 0.001). When stratified by SART ages, euploidy rates were significantly higher when comparing AI 2.0 to subjective NGS in the < 35 and 35–37-year-old categories. Euploidy rates when comparing AI 2.0 to subjective NGS were increased in the 38–40, 41 and 42-year-old categories but were not found to be significant. The euploidy rate was decreased when comparing AI 2.0 to subjective NGS in the > 42-year-old category but also lacked sufficient cohort sizes.

**Fig. 2:**
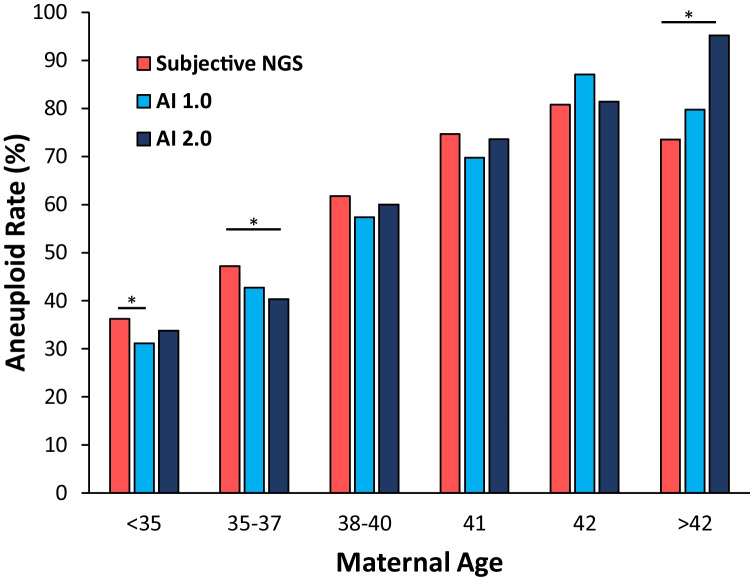
Aneuploidy rates by subjective NGS vs. AI 1.0 and AI 2.0. ^*^p < 0.05 vs. subjective NGS

Overall, the aneuploidy rate was significantly lower when comparing AI 1.0 to subjective NGS (52.1% vs. 57.0%, *p* < 0.001). When stratified by SART ages, aneuploidy rates were lower when comparing AI 1.0 to subjective NGS in the < 35, 35–37, 38–40, and 41-year-old categories with significance only in the largest cohort of < 35 (Fig. 2). Aneuploidy rates were increased when comparing AI 1.0 to subjective NGS in the 42 and > 42-year-old categories. On the aggregate, the aneuploidy rate was lower when comparing AI 2.0 to subjective NGS (54.8% vs. 57.0%) but did not reach significance (*p* = 0.238). When stratified by SART ages, aneuploidy rates were lower when comparing AI 2.0 to subjective NGS in the < 35, 35–37, 38–40, and 41-year-old categories with significance only in the 35–37-year-old cohort (Fig. 2). Aneuploidy rates were increased when comparing AI 2.0 to subjective NGS in the 42 and > 42-year-old categories.

**Fig. 3:**
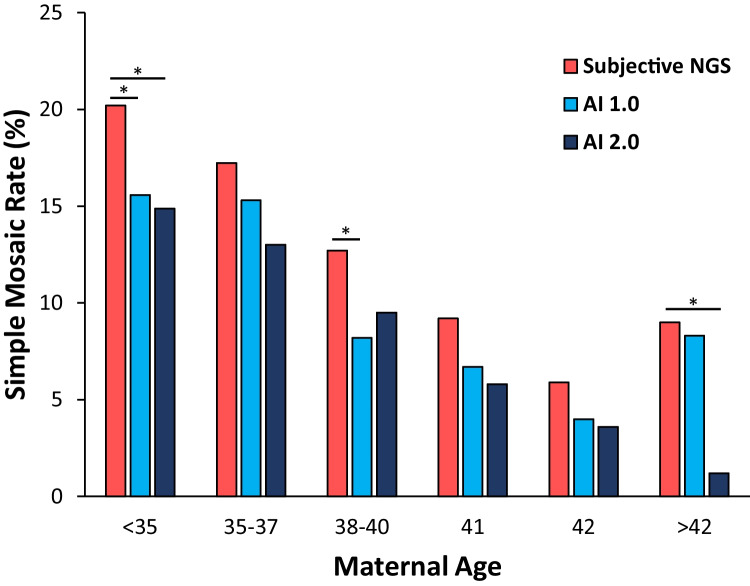
Simple mosaicism rates by subjective NGS vs. AI 1.0 and subjective NGS vs. AI 2.0. ^*^p < 0.05 vs. subjective NGS

Overall, simple mosaicism rate was significantly lower when comparing AI 1.0 to subjective NGS (11.3% vs. 14.0%, *p* = 0.013). Simple mosaicism rates were lower when comparing AI 1.0 to NGS in every SART category with significance in the < 35 and 38–40-year-old categories (Fig. 3). Overall, the simple mosaicism rate was significantly lower when comparing AI 2.0 to subjective NGS (10.1% vs. 14.0%, *p* < 0.001). Simple mosaicism rates were also lower when comparing AI 2.0 to NGS in every SART category with significance in the < 35 and > 42-year-old categories.

The triploidy rate for 69,XXY phenotype was significantly higher when comparing AI 1.0 to subjective NGS (1.1% vs. 0.3%). Triploidy rates for 69,XXX were 0% in both AI 1.0 and subjective NGS. The triploidy rate for 69,XXX phenotype was significantly higher at 0.8% when comparing AI 2.0 to subjective NGS.

### Secondary outcomes

A total of 1,174 embryos were transferred. Four hundred and five were subjected to analysis by subjective NGS, 584 via AI 1.0, and 186 embryos via AI 2.0. In the subjective NGS group, two cycles were excluded due to incomplete demographic or baseline clinical data, two cycles were eliminated due to ectopic pregnancies, and two cycles were eliminated due to elective termination, leaving 399 transfer cycles available for analysis. In the AI 1.0 group, one cycle was eliminated due to incomplete demographic or baseline clinical data, one cycle was eliminated due to ectopic pregnancy, and two cycles were eliminated due to elective termination, leaving 580 cycles available for analysis. In the AI 2.0 group, one cycle was eliminated due to incomplete demographic or baseline clinical data, two cycles were eliminated due to ectopic pregnancies, and one cycle was eliminated due to heterotopic pregnancy, leaving 182 cycles in this group available for analysis.

**Fig. 4:**
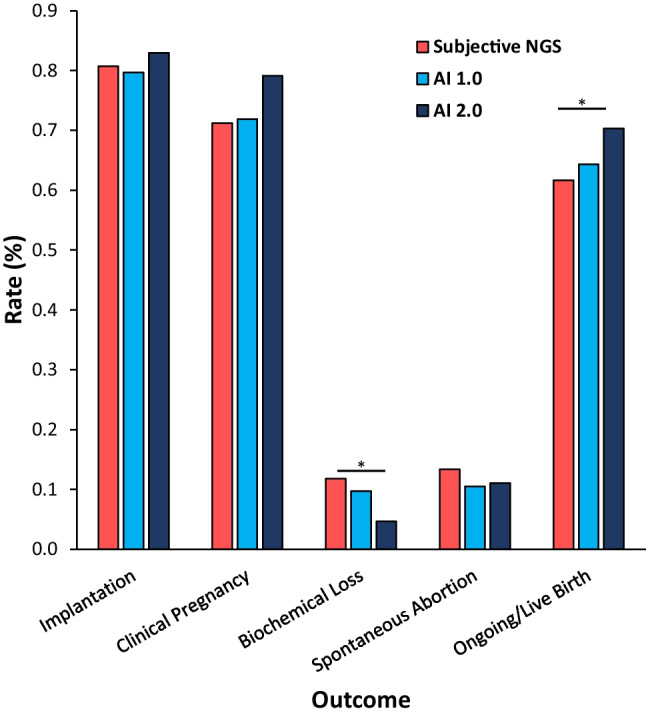
Pregnancy outcomes utilizing either AI 1.0 or AI 2.0 compared to subjective NGS. ^*^p < 0.05 vs. subjective NGS

Pregnancy outcomes of embryos tested using AI 1.0 vs subjective NGS were compared (Table 3 and Fig. 4). The OP/LBR was higher in the AI 1.0 group vs. subjective NGS (373/580 [64.3%] vs. 246/399 [61.7%]) but not significantly so (*p* = 0.419). The BPR was lower in the AI 1.0 group vs NGS (45/462 (9.7%) vs. 38/322 (11.8%) but did not reach significance (*p* = 0.409). AI 1.0 vs. subjective NGS showed no significant difference in IR (462/580 (79.7%) vs. 322/399 (80.7%); *p* = 0.744), CPR (417/580 (71.9%) vs. 284/399 (71.2%); *p* = 0.829), and SABR (44/417 (10.6%) vs. 38/284 (13.4%); *p* = 0.282).

**Table 3:** Pregnancy outcomes of single thawed euploid embryo transfers. Statistical *p* values, odds ratios, and respective 95% confidence intervals reflect either AI 1.0 or AI 2.0 compared to subjective NGS

		Subjective	AI 1.0	AI 2.0	AI total
Implantation (hCG + /embryos transferred)	Positive	322	462	151	613
	Total	399	580	182	762
	%	80.7%	79.7%	83.0%	80.4%
	*p*	-	0.744	0.566	0.381
Clinical pregnancy (sac + /embryos transferred)	Positive	284	417	144	561
	Total	399	580	182	762
	%	71.2%	71.9%	79.1%	73.6%
	*p*	-	0.829	0.053	0.565
Biochemical loss (sac-/hCG +)	Positive	38	45	7	52
	Total	322	462	151	613
	%	11.8%	9.7%	4.6%^*^	8.5%
	*p*	-	0.409	0.012	0.381
Spontaneous abortion (loss + /sac +)	Positive	38	44	16	60
	Total	284	417	144	561
	%	13.4%	10.6%	11.1%	10.7%
	*p*	-	0.637	0.877	0.381
Ongoing/live birth (loss-/embryos transferred)	Positive	246	373	128	501
	Total	399	580	182	762
	%	61.7%	64.3%	70.3%^*^	65.7%
	*p*	-	0.419	0.0497	0.381

^*^p < 0.05 compared to subjective

Outcomes of embryos tested using AI 2.0 vs. subjective NGS were compared (Table 3 and Fig. 4). The OP/LBR was significantly higher in the AI 2.0 group vs. subjective NGS (128/182 (70.3%) vs. 246/399 (61.7%); *p* = 0.049). The BPR was significantly lower in the AI 2.0 group compared to subjective NGS (7/151 (4.6%) vs. 38/322 (11.8%); *p* = 0.012). The CPR was higher in the AI 2.0 group compared to subjective NGS (144/182 (79.1%) vs. 284/399 (71.2%)) but only approached significance (*p* = 0.053). AI 2.0 vs. subjective NGS showed no significant difference in IR (151/182 (82.9%) vs. 322/399 (80.7%); *p* = 0.566) or SABR (16/144 (11.1%) vs. 38/284 (13.4%); *p* = 0.542).

## Discussion

This retrospective cohort study demonstrates that implementation of AI 2.0 significantly increases OP/LBR and significantly decreases BPR when compared to subjective NGS for PGT-A. Additionally, AI 1.0 improves OP/LBR and BPR but without significance. These advancements are likely secondary to increased accuracy in detecting euploidy, aneuploidy, simple mosaic and triploidy embryos.

Traditional computer programing used fixed formulas to perform calculations on presented data using preconceived notions or assumptions. Machine learning, an application of AI, uses programs that can adapt to training data and in turn develop more nuanced predictive classifiers. The algorithms work towards finding the most meaningful patterns to identify clusters and then use reinforcement learning to lead toward probabilistic, automated decision-making. In the most complex settings, AI can manage a continuous stream of input and feedback in a changing environment [5]. Its history began with McCulloch and Pitts in 1943 when they published a calculus supporting the concept that thinking experience alters future thinking. In 1954, Farley and Clark developed a calculator supporting self-organizing algorithms. Many others contributed, but slowly, as progress was dependent on maturing computer capabilities. It was not until 1997 that a machine outplayed a human in chess though artificial intelligence. Eventually, we reached today’s versions of AI and neural network science. Modern applications include facial recognition, automobile auto driving, and interplanetary navigation [6].

AI technology has made advances into the arena of the reproductive sciences; at the time of this writing, a MEDLINE line keyword search for AI and IVF retrieves 75 results. Machine learning was first applied to IVF by Kaufman et al. in 1997 to predict IVF outcome based on clinical and IVF lab data [7]. Since then, AI systems have been applied to ovarian stimulation, embryonic growth and development, time lapse imaging, and sperm selection [8]. However, we have not yet realized the maximum potential benefit of AI. The accuracy of machine learning in predicting embryo growth [9] or implantation potential [10] remains too low for clinical use, with accuracies and AUCs in the 0.60–0.80 range [11]. Tran et al. recently reached an AUC of over 90% using video data taken from over 10,000 embryos. However, no embryo was excluded from the analysis; most of the embryos were of poor quality, increasing the accuracy of a nonviable designation [12]. Another study applied AI to predict aneuploidy based on a single embryo image, with an AUC of 0.7, encouraging but again not high enough for clinical use [13].

AI in PGT-A has the promise to improve clinical outcomes by more accurately segregating embryos into their proper categories: euploidy, aneuploidy, simple mosaicism or triploidy. Molecular identification of mosaicism in an embryo biopsy relies on inference, interpreting intermediate copy number variation as mosaic given the logical assertation, or lack of demonstrated biological mechanism, that cells cannot contain a non-integer copy number of chromosomes. As an example, a PGT-A result might indicate 2.6 copies of chromosome 21. Given that it is impossible for a cell to contain 0.6 copies of a chromosome, it is inferred that 60% of the cells are trisomy 21, and the remaining 40% are diploid for chromosome 21. In a 5-cell biopsy, 3 would be trisomic (9 total copies), and 2 would be diploid (4 total copies), and when the 13 copies of chromosome 21 are normalized to 5 genomic equivalents, a final assessment of 2.6 copies is produced. It is important to note, however, that mitotic nondisjunction would typically produce one daughter population with a gain of material and another population with a loss of material. Thus, a viable alternative derivation of 2.6 copies would be a scenario in which 80% of cells contained a gain/trisomy while 20% of the cells contain a loss/monosomy. Therefore, we urge caution in drawing conclusions from biopsy mosaicism quantification. Similarly, it may be tempting to focus on the direction of change, for example, speculating that a mosaic monosomy presents a lesser risk, when in fact, a trisomic population may exist elsewhere (e.g., inner cell mass) in the embryo.

High-throughput pattern recognition has allowed clinics to become more confident about the competency of mosaic embryos which has remained a clinical dilemma since the very early days of PGT-A using aCGH [14]. While mosaic embryos are known to have reduced pregnancy potential, their transfer commonly results in normal live births. Greco et al., also testing with aCGH [15], published a case series reporting six live births following 18 transfers of mosaic embryos, some with multiple mosaic abnormalities. Maxwell et al. [16] used subjective NGS to re-analyze biopsy specimens originally tested via aCGH. They found that 15.8% of normal live births diagnosed as normal via aCGH were found to be mosaic when tested via NGS. A multicenter analysis by Munne et al. [17] found that when using subjective NGS, 41% of mosaic embryos transferred in their study led to ongoing pregnancies. It is now established that there are varying degrees of mosaicism, ranging from a single abnormality to the involvement of multiple chromosomes known as complex mosaicism. Munne et al. [17] also determined that complex mosaic embryos have significantly lower implantation rates (10%) than single whole chromosome mosaic, double whole chromosome mosaic, or segmental mosaics (50%, 45%, and 41%, respectively). Thus, while an enormous effort is being put forth to unlock the mysteries of mosaicism, it remains problematic and a primary area of interest. AI platforms add to the collection of small pieces working to solve the larger puzzle.

Despite significant strides made, as is the case in all analytical systems, PGT-A is not immune to variability in signal-to-noise ratios. Both exogenous and intrinsic sources of assay noise can be problematic, as this noise has an impact on signal quantification that is similar to what is observed when attempting to resolve a mixed population such as mosaicism. This phenomenon makes accurate identification of embryo biopsy mosaicism rather challenging, particularly when utilizing subjective human interpretation. Even when highly experienced scientists use sophisticated chemistry and state-of-the-art sequencing instruments, there are few perceptible indications that differentiate the signal of a mixed population from noise. Herein lies the strength of machine learning. When additional information is available for training, such as when the data from a sample belongs to an embryo that produced a healthy live birth, or alternatively one that exhibits an imbalanced inheritance of a structural rearrangement with known and specific breakpoints, it can be leveraged to teach machine learning algorithms to decipher the difference between true signal and noise patterns that have been observed in a large population of known negative samples.

There are several other approaches that, alone or in synergy with artificial intelligence, can be utilized to optimize PGT-A results by either increasing true signal or decreasing noise. Though not impervious to noise, the most intuitive of these measures is to increase the number of observations made when assessing the three billion base pair human genome. We have seen PGT-A evolve over the years toward an increase in measurements or datapoints. Generically speaking, the progression through FISH, qPCR, aCGH, targeted sequencing, and SNP array has taken us from a few, a few dozen, a few thousand, tens of thousands, and hundreds of thousands of datapoints, respectively. PGT-A by subjective NGS, topping the one million data point mark, has been praised within the PGT-A community for years for producing the cleanest data of all these sources. The AI 2.0 system, described here for the first time, has pushed things even further to four million reads, both increasing the number of genome observations and feeding the AI algorithms. Importantly, it should be noted that even at this read depth, using a 36 base pair read length yields only 144 million bases covered, or just under 5% of the genome, suggesting that there remains plenty of room for deeper interrogation.

Over a decade ago, SNP microarray technology became popularized for use in PGT, proven to be capable of identifying aneuploidy and translocations in blastocyst biopsy specimens [18]. Its value in detecting embryonic polyploidy in human embryos was later substantiated [19]. SNP microarray technology has taken on an important role diagnosing ploidy in perinatal genetic diagnostics [20]. The stochastic nature of chromatin structure and DNA amplification means that an aggregate 0.05 × genome coverage translates to thousands of loci with sufficient depth to investigate whether an allele is homozygous or heterozygous. While it is of insufficient depth and accuracy for genotyping, low-pass heterozygosity assessment can play an important role in PGT-A. In a typical diploid heterozygous allele, there is typically one copy of the major isoform (A) and one copy of the minor isoform (B). The 1:1 relationship yields a minor allele ratio of 0.5. In the case of a monosomy or haploidy, there is a loss of heterozygous alleles, manifesting 0.0 or 1.0 minor allele ratios. In the context of trisomy or triploidy, heterozygous alleles can be either AAB or ABB, producing minor allele ratios of 0.33 or 0.67. Utilizing the aneuploid-derived drift in heterozygous ratios, as is done here in AI 2.0, serves as an additional layer of information. These ratios can be used to either confirm or reject putative copy number variations, and importantly, identify haploidy and triploidy. Further, if parental DNA is available for trio analysis, SNP inheritance patterns can be utilized for determining which gamete is the source of meiotic aneuploid. Secondary copy number assessment, detection of haploidy and all forms of triploidy, and determination of the origin of aneuploidy are all SNP-dependent capabilities that are not possible via standard subjective NGS. Optimization of analytical accuracy should yield minimal false-positives (more euploids) as well as minimal false-negatives (yielding lower rates of adverse outcomes), both of which were observed here.

## Strengths

Our strengths include sample size with 4,765 retrieval cycles and 24,908 embryos being utilized for primary outcome analysis and 1,174 embryos transferred via STEET analyzed for secondary outcomes. This study was done at a single center providing increased homogeneity among embryologist, culture variables, and medical protocols. To the best of our knowledge, this is the first report to assess the impact of the utilization of AI in PGT-A. Finally, both embryo biopsy and transfer data were studied which allows for speculation as to why OP/LBR increased and BPR decreased.

## Limitations

The retrospective nature of this study is a limitation, and not all treatment or diagnostic attributes could be controlled for. Given the nature of the study design in addition to emerging and evolving technologies, not all groups had equal or comparable cohort sizes, particularly in the newest technology and older SART age groups. Specifically, the discrepancy between duration and the number of outcomes in the subjective NGS cohort can be attributed primarily to the fact that two PGT-A technologies were used during this period: aCGH and subjective NGS. In contrast, with integration of AI technologies, one hundred percent of cases were run on the respective technology that was clinically available to patients at that time. Additionally, PGT-A utilization rates increased significantly as the value of the technology became increasingly apparent. While trends here are encouraging, they will have to be revisited when more data becomes available. Additionally, the clinical outcome of embryos with more than two mosaic findings was not independently studied because these were assigned to the category of abnormal, therefore, not suitable for transfer. Although there is evidence that complex mosaic embryos have decreased implantation potential, information on this subject is limited. In addition, uterine factor or history of multiple pregnancy losses were not incorporated into this analysis. Furthermore, product of conception testing was not done routinely or data unavailable, which would provide valuable insights into potential false-negative rates (and differences) between technological approaches. Similarly, aneuploid embryos were not transferred, precluding the ability to determine the true false-positive rates of any of the technologies. Finally, due to small cohort sizes, age stratification of pregnancy outcomes was not performed. While it is possible that age stratification could modify interpretation of the data, comparable pregnancy outcomes have been shown across maternal age with euploid embryo transfer [21].

In conclusion, this retrospective cohort study found that the implementation of AI 2.0 significantly improves OP/LBR and significantly decreases BPR when compared to subjective NGS for PGT-A. This is most likely due to increased accuracy of AI 2.0 in detecting euploidy, aneuploidy, simple mosaicism and triploidy embryos as seen in our results. Given that artificial intelligence generally produces stronger classifiers with increased data, larger, randomized and prospective studies are needed to confirm these findings.
